# A quintuple helix model for foresight: Analyzing the developments of digital technologies in order to outline possible future scenarios

**DOI:** 10.3389/fsoc.2022.1102815

**Published:** 2023-01-25

**Authors:** Elvira Martini

**Affiliations:** Department of Law, University “G. Fortunato”, Benevento, Italy

**Keywords:** future, foresight, forecast, digitalization, organizations, quintuple helix model, triple helix

## Abstract

The challenge of contemporary society is that of planning possible paths for the future. In the current scenario of hyperconnection, men and technologies and human and artificial intelligence are intertwined in such complex ways as to generate multiple possible futures up to the limit of the capacity of imagination. In particular, it is precisely the frontier of digital and technological changes that obliges social actors and socio-economic institutions to know how to intercept the dynamism of the transformations taking place, supporting the ability to imagine a desirable future, which goes in the intelligent direction of sustainability, of wellbeing and the ethical responsibility of one's actions. In this perspective, the reflection on the so-called future studies is inserted, which becomes a necessity, especially in times of change: If the rhythm of change increases, we need to look further, but future studies are also a philosophy of thought because the future is already part of our present life in the form of anticipation of the future; and this is all the more true as social changes are improvised and systemic complexity increasingly turbulent. Based on these statements, this study aims to analyze how the triple helix model—or rather the quintuple helix model—can be a reference paradigm for social and technological forecasting in a systemic attempt to look at the future of science, digital technology, society, economy, and their interactions, in order to promote social, economic and environmental benefits. From the social perspective, the model could provide guidance to improve the anticipatory profile of organizations and communities, helping to understand—in a short time—what the present actions will be: Predict, discover, and anticipate united in active participation, communication, knowledge, and action become so essential in the processes of production, as in the past it was the accumulation of capital, and also the ethical sensitivity begins to play an increasingly critical role.

## 1. Purpose of the study

To face the social, economic, and cultural effects of the pandemic, social and political institutions around the world have been investing all available resources for 2 years and working on the implementation of recovery plans and tools (think Next Generation EU) to overcome the crisis and make the planet greener, more digital, and more resilient.

In this frenetic project of rebirth, there is a greater effort and a greater will to ensure a better future for the next generations; at the same time, however, there also emerges a bitter reflection on the fact that the pandemic has caught us completely unprepared, discovering all our fragility.

We, therefore, need to ask ourselves about the mistakes that have been made so far and the lack of reflection with regard to the consequences of our actions on the environment, the misuse of technology, consumption, and waste.

These questions, and many others, re-propose the need to think about the future in a long-term perspective, picking up the signals and helping to design more resilient policies in the face of systemic shocks: In other words, today more than ever, it is necessary to invest systematically and synergistically in future studies (Arnaldi and Poli, 2012; Poli, 2019, 2020a,b,c).

Future studies become a necessity, especially in times of change (Barbieri Masini, 2012): If the rhythm of change increases, we need to look further (Berger, 1964), but future studies are also a philosophy of thought because the future is already part of our present life in the form of anticipation of the future, and this is all the more true as social changes are improvised and systemic complexity increasingly turbulent (Luhmann, 1990).

Based on these considerations, this study aims to analyze how the triple helix model (Etzkowitz and Leydesdoff, 1995, 1998, 2000, 2001, 2003; Etzkowitz, 2004, 2008; Leydesdorff, 2005, 2006, 2008, 2010, 2013, 2021)—or rather the quintuple helix model (Carayannis and Campbell, 2009, 2010, 2012; Carayannis et al., 2012, 2016)—can be a reference paradigm for social and technological forecasting, in a systemic attempt to look at the future of science, technology, society, the economy, and their interactions in order to promote social, economic, and environmental benefits (Tegart, 2003).

From a social perspective, the model could provide guidance to improve the anticipatory profile of organizations and communities, helping to understand in a short time what the consequences of the present actions will be.

From the point of view of technological application, the challenge is in understanding possible technological developments and in outlining possible economic, social, and market impact scenarios.

In particular, the misuse of technology, the digitization of many living and learning environments, and the collection, management, and analysis of huge data raise many ethical and social issues; concerns about data security and privacy have been highlighted, as well as the more substantive issues about the influence and control that these technologies can have on people's lives. These risks can undermine citizens' confidence in these technologies and in the institutions and companies that use them (Amaturo et al., 2022, p. 636).

The systemic structure of the model, its internal dynamism, the monitoring action, and the feedback mechanism—without which the same general system would disappear, so much so as to talk about *teleonomy of the system* (Paparella, 2014)—are the basic preconditions for talking about foresight spaces and finding stable systemic solutions to very complex situations (Dubois, 2000; Leydesdorff, 2008, 2021). The model thus structured, in short, would help to strengthen the contribution already made by the Science and Technology Studies on the need to apply the paradigm of co-evolution between society, science, and technology: The change, in fact, is never only technical or social but is always socio-technical, that is, resulting from the interaction between the social sphere and the techno-scientific sphere (thus highlighting the problem of the ethical technology assessment).

An overview, albeit brief, of the impotence of future studies will be offered below, followed by a description of the operation of the triple helix and quintuple helix model, trying to use the model as a possible driver to favor not only spaces of knowledge and innovation but also foresight and in particular technology foresight: The challenge is to systematically organize reflection on possible future scenarios to anticipate or prevent the most impactful consequences from an ethical–social point of view.

## 2. The importance of thinking about the future

Although the reflection on the future is a constant in human history, the systematic attention toward the study of the same develops starting from the 50s and 60s of the last century, undergoes an interruption with the energy crisis at the beginning of 1970 and relives a new impetus (which still lasts today) in the late 70s and early 80s (Arnaldi and Poli, 2012). With the awakening of attention to this type of study and research, their contribution has been underlined not so much in the prediction of specific events as in the possibility of indicating alternative paths toward the future.

This is an opinion not shared by all scholars but supported instead by the intellectuals of developing countries: The latter believe that thinking about the future is a means of overcoming the present and giving life to a different world closer to one's expectations. It is an approach that leads to making value choices that can be different in each culture (Kothari, 1975).

The complexity of the available alternatives is closely connected to the existence of different values based on different cultures and, consequently, to the presence of divergent worldviews. “[…] The possibility that the future is actually open depends simultaneously on the acceptance of belonging to a culture, including the set of values that characterize it, and the recognition of a plurality of cultures and visions of the future (Barbieri Masini, 1982, 1994)” (Arnaldi and Poli, 2012, p. 14, *my translation*; Facioni, 2019).

As Barbieri Masini (2012, p. 13–22) explained well, thinking about the future responds to three main motivations that continuously intertwine with each other: Thinking about the future is a need, thinking about the future is a choice, and thinking about the future is a way of structuring our way of thinking.

Thinking about the future is a need that makes itself felt, especially in times of great change. Berger (1964), supported “the importance of studying the future using the image of a car launched at great speed: the higher the speed, the farther the headlights must illuminate in order to avoid obstacles” (Barbieri Masini, 2012, p. 14, *my translation*).

Thinking about the future and studying possible futures also corresponds to a choice that every person, or society, must make in the present. Talking about choice means asking questions: Is it right to worry about the future? Should we worry about the consequences of our actions in the future and the impact that predictions about this same future can have on the present? Or, since the future is unknown, is it not better to worry only about the present? Precisely, this last question poses a central problem of reflection on the future: While aware of living in a world resulting from revolutionary changes, we continue to see the future as something essentially similar to the present. “It is a clear cognitive illusion: *we know* that things have changed enormously, but *we believe* that tomorrow will not be so different from today” (Poli, 2019, p. 7, *my translation*).

Here, we come to the third motivation that the challenge of looking at the future through studies is also that of turning it into a way of thinking that shapes our soul and allows us to give meaning to our lives, our daily actions, and our decisions. Therefore, to overcome that cognitive obstacle which resides in the incapacity of common sense to intercept the future. Thinking about the future and glimpsing today what will happen tomorrow means getting out of the tranquility of repeating the ordinary and accepting to consider other possibilities (Poli, 2019) that are already part of our life in the form of anticipation of the future.^1^

### 2.1. Evolution of future studies

It was 1943 when the german Flechtheim inaugurated future studies with the term *Futurologie*, with the aim of eliminating wars in favor of a peaceful state, stabilizing population growth, eliminating hunger, misery, oppression, and the exploitation of natural resources (Flechtheim, 1943; Poli, 2012).

Berger is another author who deserves to be mentioned and who is considered to have developed the French version of social forecasting: *perspective*. Berger notes that the constant acceleration of technological and social changes weakens any effort to extrapolate forecasts, starting from the analysis of previous situations. His great contribution was to understand the importance, not so much of how to better predict the future as of how to prepare to face an ever-changing world and how to choose the actions capable of leading us toward the objectives we consider most preferable (Cournand and Lévy, 1973). The transition from forecasting in the strict sense to preparing for the challenges of the future shifts the emphasis from the greater or lesser correctness of the forecasts to the ability to face up to new developments and realize the values that are considered significant. Forecasting as an extrapolation from the past is no longer sufficient and is being replaced by the ability to orient oneself in real-time, to choose reference values, and above all, to make proactive decisions aimed at implementing the desired changes. For Berger, the future was hinged on the present.

Another important contribution dates back to the 1960s with the studies of De Jouvenel (1967), who introduces the distinction between *facta*^2^ *(facts)* and *futura* (see text footnote^2^) *(futures)* and argues that noting that the sciences deal with facts, respect to which information and data can be collected. Anything that has not happened yet, anything in respect of which there is no data to be analyzed, does not fall within the purview of science. In other words, this means that there is no science of the *futura* and if *facta* are elements of reality, the *futura* is nothing but unreal.

Bell's introduction of the category of “disposition” makes De Jouvenel's distinction between *facta* and *futura* less elementary (Bell, 2003, p. 76). This is a significant step forward because dispositions, unlike the *futura*, do not have the nature of cognitive artifacts, but are real facts: Those facts that could occur if the right circumstances were to occur.

In this sense, then the *futura* becomes a particular type of fact, that is, those facts that are possible even if, at the moment, they are not actual. The dispositions that interest future studies are obviously not so much dispositions of a physical nature but dispositions as the ability to change on the part of individuals, groups, and entire societies. The key to accepting the need to study futures is knowing how to consider these capacities as real, whether they have already been expressed by some effective transformation or are still latent and possibly ready to manifest themselves when the conditions are ripe. Even if not all possible futures have the nature of dispositions, the fundamental step forward made by Bell helps to understand that future, present, and past are linked together, that there are structures that connect them, and that they exist even when they are not explicitly active. Not all of reality is fully unfolded before our eyes; there is also a reality that exists but is not yet operational.^3^

Among other things, relationships and social events are bound by a series of rhythms of different natures and duration, not only directly social but also physical and biological. Whether visible or not, natural rhythms form the background from which social connections and relationships emerge that also present aspects that vary from the perfectly visible to the totally implicit. The different rhythms do not proceed in isolation from each other but interact in many different ways. Even if we cannot change the physical laws of nature, we are nevertheless able to exploit them to our advantage: When we build a road, a bridge, or when we divert a river, we change nature by using the laws of physics to our advantage; when we select and modify the functioning of fruit trees, we alter nature by using the laws of biology to our advantage, or, at least, we think we do it to our advantage (Poli, 2012, p. 31–32).

In light of what has been previously briefed,^4^ it can be concluded that the three main areas of the theory of the future are as follows: predictions, discoveries, and anticipations. In his aforementioned book *Lavorare con il futuro*^5^ Poli (2019), Poli clearly illustrates the differences between these dimensions (Poli, 2019, p. 11–18). Forecasting activities, in the strict sense, include the use of different types of formal models that provide indications of the progress of certain trends (inflation, the unemployment rate, climate change, etc.). The forecasting models work in light of some possible fundamental assumptions that condition their validity. For their creation, we rely on the behavior of variables considered decisive in the definition of a certain area, which guarantees reasonable forecasting reliability.

The most evident limitations of econometric type forecasts, for example, are represented by the fact that these forecasts work for rather short time windows together with the further constraint which corresponds to what the author calls the principle of continuity: “the idea that the system we are talking about, will continue to function much as it has until now” (Poli, 2012, p. 12, *my translation*).

The same argument can be made for other types of forecasts, such as climate forecasts which instead adopt very long time windows^6^ of up to 100 years; also, in this case, there is a basic assumption that, as before, corresponds to a principle of continuity: It is the constancy taken for granted of the laws of physics.

These first two types of models are both serious, robust, and solid, anchored on sophisticated scientific and methodological knowledge. The problem is that one might also be interested in intermediate time windows and discontinuities, surprises, and novelties.

When dealing with intermediate windows and changes (but also with qualitative data and unreliable information), it is necessary to take a different perspective.

This is how we go from prediction to discovery.

Understanding the distinction between futures in the singular and futures in the plural is the first step in understanding how predictions and discoveries differ. First of all, it is necessary to start from the awareness that something can always be done to try to understand how events could develop; it is possible to discover some possible futures that help keep in mind different ways in which reality could be articulated. Central in this circumstance is, therefore, the passage from the singular of future (almost as if it were our only possibility before us) to the plural of future (things can go in different ways, if we see them, we can try to prepare ourselves and not get overwhelmed from the news).

The moment we move from an implicit idea of the future to an explicit idea of the future, the value of the same discovery emerges: Seeing the ways in which situations can evolve allows us to try to prepare ourselves, but the clarification of the possible futures linked to their discovery also has a clear ethical relevance: If social actors, institutions, and organizations see what could happen, they cannot then avoid taking responsibility for what they will or will not do.

In summary, the difference between forecasting and discovery lies in the constructive modality adopted to make futures explicit: In the case of forecasting, *futures* are constructed as repetitions of past experiences, while in the case of discovery, futures also contain authentic innovations and discontinuities (Derbyshire and Wright, 2017; Tuomi, 2019). The third dimension of future theory concerns the question of how to translate models into decisions and actions. This node is very critical as a future exercise that does not translate into practice is a failed exercise. Therefore, translation into action is not incidental.

The anticipation consists of two elements: A modern one and its translation into action. Forecasts are nothing more than a model and only tell us what could happen; seeing what might happen and changing one's behavior accordingly is a case of anticipated activity. Certainly, the best-known definition of anticipation is the one proposed by Rosen (1985), according to which an anticipating system is a system that contains a predictive model of itself and/or its environment, in such a way as to be able to take his decisions in the present moment in light of the prediction that something could possibly happen in the future.

Anticipatory behavior is more robust than reactive behavior. Reactive strategies are often expensive and inefficient (consider all the costs incurred to stem the COVID-19 pandemic^7^). Therefore, every single actor and even more every single economic, political and cultural organization needs to reflect and understand that there are many different ways of anticipating because it is necessary to find from time to time the most suitable ones for one's situation, understanding the cognitive and social constraints that filter and condition the translation into action of a model.

In all types of reality, the phenomenon of anticipation is widespread. All varieties of life have an anticipatory character: society and its structures are anticipatory; the brain and the mind work in advance, and even some of the non-living and non-biological systems can be anticipatory. For this reason, studies on anticipation have been carried out in numerous disciplines ranging from philosophy, physics, biology, psychology, semiotics, and social sciences. With particular reference to the latter, it should be remembered that Schütz (1960) developed applied phenomenological optics to the social sciences. For the Austrian scholar, we do not live simultaneously in different contexts of meaning: the thematic system, the interpretative one, and the motivational one. Due to the way the motivational system works, actions are typically structured by two kinds of opposition: that between my actions and the actions of others and that between future and past actions. Future actions are interpreted according to the key “in order to”, while past ones are understood according to the key “because of”. The former are elements that shape the action from within, while the latter requires reflexive acts on actions already carried out.

Riegler (2003) and Leydesdorff (2008, 2021) (the latter, together with Etzkowitz, developed the triple helix model) have applied the idea of anticipation to social systems. Actions are always elements of larger projects which in turn draw on different reservoirs of knowledge. One of the most familiar components of knowledge is the reservoir of typical expectations, which can become actual under typical circumstances and predetermine typical actions.

Social systems often try to cope with a new situation by replacing its details with familiar activities and behavioral structures that show a high degree of predictability, to try to keep the situation under control, to be able to anticipate its outcome; in this sense, therefore, even new experiences can be familiar with respect to their typology.

In the last 30–40 years, important experimental data have demonstrated that all the axioms of the expected utility theory have been violated by real subjects in experimentally controlled situations (Berthoz, 2004). Agents are not ideal or idealized decision-makers but, on the contrary, real agents who can make mistakes, for many reasons: social pressure, the tendency to agree with others, the influence exerted by hierarchical structures, the role of emotions, the desire to be right, the way problems are represented.

All this suggests the importance of updating programs on how to make decisions, especially if these concern education, the economy, digitization, and investment in technology.

### 2.2. Technological choices and anticipation

Technological forecasting has experienced real global flourishing in recent decades. If in the 50s and 60s of the last century, this type of forecasting activity found its fundamental justification in need of the Defense Industry and had its main promoter in the Rand Corporation; starting from the 70s, it is the economic sector and justifying and stimulating the implementation of forecasting activities, in support of industrial planning (Arnaldi, 2012).

Today the techniques of anticipation that have science and technology as their object are promoted to respond to some trends of growing importance that characterize the global economic vision. In contrast, the increase in competitive pressure on organizations and territories, the growing budget constraints which limit public expenditure, the consequent need for a more efficient allocation, the public dimension of science, the ethical consequences of the application of technology or the excess of digitization (Martini and Vespasiano, 2017); all this makes scientific knowledge and technological developments the subject of debate and criticism. Conversely, the elaboration of policies must take into account the increase in the complexity of decisions caused both by factors of a general scope such as the interaction between systemic levels, the diversification of the actors involved in the elaboration and implementation of policies, and by specific characteristics of different technical-scientific fields, such as the integration between different technologies and the emergence of multi- and inter-disciplinary scientific fields (Martin, 1995; UNIDO, 1999; Tegart, 2003), the interdependence and existing trade-offs between different policies, the pervasiveness of technology in all areas of human life (Grupp and Linstone, 1999), the danger of the so-called technological singularity, that is, that point where technological progress accelerates so much as to overcome the ability to understand, control, and predict typical of humans (Kurzweil, 2005).

All these issues highlight how over time, there has been a gradual loss of centrality of the technical dimension in favor of increased attention to the context, understood in the broadest sense, of innovation. For some time now, the term *Future-Oriented Technology Analysis* has identified and summarized this change and the broadening of the horizon (Cagning and Keenan, 2008).

Based on the considerations just expressed, the following pages will be devoted to the analysis of the triple helix model (as a driver of socialization of innovation and knowledge). We will then pause to analyze how the same model, in its extension to five helices, can stand as a candidate for supporting change and broadening horizons, favoring the creation of an increasingly *Future-oriented Technological Analysis*, demonstrating the importance of future studies and the ways in which we can develop the information needed to shape it.

## 3. A space of knowledge and foresight: The triple helix model

“The research and innovation system has undergone profound changes of an organizational, sociological, and managerial nature over the last century, particularly in the most industrialized countries, finding itself interacting more and more strongly to promote knowledge and economic development. The academy, not always voluntarily, has progressively permeated itself with values, organizational models, and social roles typical of the entrepreneurial and financial system, becoming a key element in innovation policies all over the world, both as a source of new technologies for start-ups and existing industries (Etzkowitz, 2008)” (Martini and De Luca Picione, 2022, p. 438). For its part, the industrial system has also re-evaluated the importance of the university, rediscovering the need to recover the leverage of academic research and the R&D sector to promote innovation and competitiveness after years in which the cost of labor, the protection of the markets or the weakness of the currency, have unfortunately represented much more effective levers of competitiveness (Calderini, 2005).

Furthermore, it is argued that the “complex relationship between the organization of knowledge and technology could be better addressed with a federal approach, that is, with the decentralization of power to universities and research centers, thereby strengthening the possibility of an evolutionary self-organization from below” (Viale, 2001, p. 58, *my translation*). As Etzkowitz says, “this involves carrying out continuous experiments on the relationship between science, industry and government, in order to find the right fields of application for the innovations of the future: the Endless Frontier model is gradually replaced by that of the Endless Transition” (Etzkowitz, 2008).

The environmental and selective constraints of the global market, in contrast, and the implications deriving from the generation of new technological and digital knowledge on the other, have increased the effect of calling for an integration between the three actors of the university, the enterprise, and of the government.

In fact, the initially bilateral relations between government and business and between university and business have transformed over time into trilateral relations of the university–business–government type, thus creating the emergence of a three-vector development model.

This relational mechanism is useful for triggering and sustaining development dynamics based on innovation and technical progress, better known as the sociological metaphor of the triple helix model (developed for the first time by Etzkowitz and Leydesdoff, 1995, 1998, 2000, 2001, 2003); moreover, it wants to be the sociological expression of a new socio-economic-political order based more and more on knowledge.

As Luhmann would say, the “social-world system” becomes more and more complex than the biological one, and just two helices are no longer enough, but the need arises to involve other actors: The latent presence of a third dimension can reduce the related uncertainty to the interaction of the first two actors.

This new model has the same elementary actors of the Sábato triangle (Hatakeyama and Ruppel, 2004) and of the National Innovation Systems (Freeman, 1987, 1995; Lundvall, 1992, 1998) but foresees a different dynamic of relations between them. In fact, the actors here are not static—since they are continually in a state of transition—and the succession of interrelations develops according to a spiral model that presents different types of relations between the public sector, the private sector, and the university, depending on the level of capitalization of knowledge.

In other words, the supporters of the triple helix highlight that the relationships that were valid in the past between government, industry, and universities are still valid today, but with the formulation of a new institutional model characterized by the presence of specific groups, who meet and confront each other to solve the new problems posed by economic and social changes and which could give a significant boost to the study of social and technological forecasting, in order to be able to anticipate its consequences in social, economic, and ethical terms.

**Figure 1:**
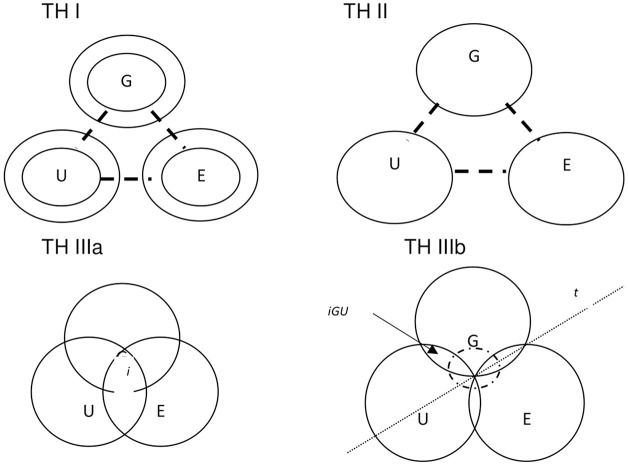
Evolution of triple helix model (TH I, TH II, TH III). Source: Our elaboration by Etzkowitz (2008) and Leydesdorff (2008). G, government; E, enterprise; U, university; i, common ground; t, time line.

The evolution of this model is through three phases, as shown in Figure 1. The premature version of the triple helix (I) can be displayed with three independent circles connected by dashed lines. The three spheres are defined as communication subsystems that interact through market operations, technological innovation, national governments, and related interfaces.

In a more developed version of the triple helix (II), the three independent circles are connected by solid lines, representing intermediary organizations such as technology transfer offices or legal offices. In this study, the three spheres are institutionally defined and maintain a certain autonomy but interact through strong, well-defined boundaries.

The advanced version of the triple helix (III) appears in the shape of three overlapping circles with the internal communicative core, represented by a small circle superimposed on the three intersecting circles. The three institutional spheres, in addition to carrying out their traditional functions, also assume the role and perspective of the others (think of universities that can play quasi-governmental roles as local organizers of innovations).

“In the Triple Helix (IIIa) configuration the three helices share a common ground indicated by i. Under certain conditions, however, this overlap can also become negative. This eventuality is represented in the Triple Helix (IIIb) configuration, where the center becomes a cavity that can be considered as a negative entropy within the system. This system works, over time (t), in terms of different communications with respect to its interfaces. When all the interfaces communicate, we can suppose the birth of a hyper-cycle that integrates the systems in a differentiated way. Integration is not unique because there is no center where it can develop; this is why, in this situation, properties of autopoiesis, selection and re-differentiation will be necessary” (Leydesdorff, 2006, p. 402, 403).^8^

On a more in-depth reading dimension, the model also postulates that “the triple helix circulation occurs on macro and micro levels. Macro circulation moves among the helices while micro circulation takes place within a particular helix. The former creates collaboration policies, projects, and networks while the latter consists of the outputs of individual helices. Lateral social mobility, the introduction of expertise from one social sphere to another, can stimulate hybridation invention and innovative of new social formats. Horizontal circulation is thus more likely to have a radicalizing effect than vertical circulation with its inherent conservative bias. Vertical circulation occurs through upward movement of individuals within an institutional sphere typically through recruitment of new persons of talent from lower strata, revivifying an elite” (Etzkowitz, 2008, p. 21).

More specifically, “the actors represent the micro level, within which the evolutionary characteristics of the model are clearly visible. The performances of the actors bring together roles and models that involve various and different cultures, previously separated: university researchers become entrepreneurs of their own technologies; entrepreneurs work within universities and related technology transfer offices; public researchers invest their time working within industries; industrial and university researchers manage regional agencies responsible for technology transfer. The meso level is represented by the institutions: it is that level that organizes production and makes use of technological knowledge. It can be divided into three sub-categories: hybrid innovation agents (high-tech spin-offs, venture capital); the innovation interfaces between businesses and research; the innovation coordinators, responsible for the coordination and management of the various phases of the innovative activity. Finally, the rules represent the macro level, which essentially has the function of guiding political incentives: the actors will make decisions in compliance with the regulatory framework and the tax incentives already available (think of the legislation on property rights)” (Viale and Ghiglione, 1998, p. 3; Martini and De Luca Picione, 2022, p. 440).

## 4. The quintuple helix model: Paradigm of future studies and digital technology foresight

As stated in Martini and Vespasiano (2015, p. 79–85), knowledge represents the key to success in sustainable development. In other words, it is increasingly convinced that nation-states that focus on the progress of society, on greater competitiveness of the economy, and on a better and sustainable quality of life, must apply the resource of knowledge. In this way, the knowledge resource is transformed into the “most fundamental resource” (Lundvall, 1992, p. 1), with the quality of a “knowledge nugget” (Carayannis and Formica, 2006, p. 152). Knowledge, as a resource, is created through creative processes, combinations, and productions in so-called “models of knowledge” or “models of innovation” and thus becomes available to society: “we can also call it the creativity of knowledge creation” (Carayannis and Campbell, 2010, p. 48). Typically, innovation models do not include an end-to-end view. Most of the innovation policy is focused on the ability to innovate and on input factors such as R&D investment, scientific institutions, human resources, and capital. Such inputs often act as a proxy for innovativeness and correlate with intermediate outputs such as patent counts and outcomes such as per capita GDP. While this type of analysis is generally indicative of innovative behavior, it is less useful in terms of discriminatory causation and what drives successful strategy or public policy interventions (Yawson, 2009).

**Figure 2:**
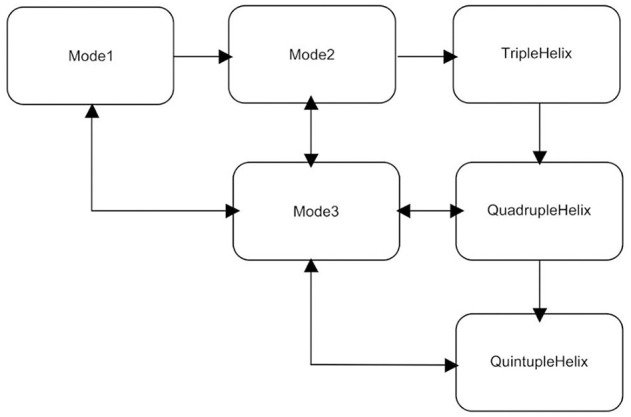
The evolution of the models of knowledge creation. Source: Carayannis et al. (2012).

This situation has led to the development of new frameworks for the innovation system, and we want to refer here specifically to the six currently existing models of knowledge creation and innovation creativity (Figure 2 and Carayannis and Campbell, 2012; p. 13–28; Carayannis et al., 2012, p. 2–3; Carayannis et al., 2016).

The previous models developed for measuring innovation are many and varied; they are linear in nature and neglect the interrelationships between the various indicators and metrics. Science investigations are often articulated in an input–output framework: Inputs are invested in research activities that produce outputs. This framework is purely an accounting framework based on the expected economic benefits of science. Indeed, most of the existing methodologies for measuring innovation are driven by research and practice in the fields of accounting, economics, human resource management, intellectual property, etc. All previous revisions of these models have focused on company-level analysis with an accounting, economic or strategic lens, and most of these models have not been applied directly to the assessment of national innovation systems. However, many empirical research studies and institutional policy frameworks refer to the key elements of these models in their conceptualization. However, the main gap identified in the literature with these new national innovation policy portfolios is the linear nature of the presentation, just like previous innovation models.

The “core model” is the triple helix which is a valid socio-economic mechanism, where the relational networks (enterprises–universities–governments) get together to produce social values from knowledge. The analytical examination of this model shows that the critical point is the quality of the social capital nets, starting point to set up, sustain and manage the triangulation we are discussing. The triple helix governance, while pushing the communication and the relationships among the territorial actors, favors the forms of collective learning and facilitates the economic and cultural exchanges necessary for the development and social inclusion. The dynamics of the triple helix want to promote an innovative atmosphere, encouraged by all actors (Leydesdorff, 2005): In fact, every actor assumes roles and tasks uninterruptedly, reformulating the agreements with the others. At the same time, these interrelations are also realized inside every single actor, reformulating continuously structures, characteristics, and objectives.

As we have already seen, “in the dynamics of the Triple Helix the role of socialization is very important; in other words, we emphasize the importance of social capital networks. In fact, social capital facilitates the diffusion of a climate of trust and this collaboration makes learning (individual and organizational) simpler and more effective, which in turn generates intellectual capital, innovation, and competitiveness. An elevated intellectual capital could create greater job opportunities, to decrease unemployment rates, and favor social inclusion (Livraghi, 2003, p. 108). A greater active participation of all the members of society to the productive processes, a smaller social exclusion could favor the formation of social capital, revitalizing and recomposing internal relational dynamics to the circuit of the Triple Helix. From there it comes a virtuous circle of development and social well-being” (Martini and Vespasiano, 2011, p. 179).

However, the triple helix model cannot be considered a closed model to apply to the perfection of any local context: It demands continuous territorial adjustments and integrations because every context has its net of the relations—weak and strong—that give form and substance to the relative social capital.

As Bagnasco (2006) explained, it is a sociological problem: The territories are various, the social, cultural, and economic pre-existence are ballast to the development programs and their various abilities to create nets of social capital, and institutionalized confidence can be powerful motors in order to accelerate the realization of development programs.

These reasons have led other authors to adopt new perspectives, imagining and proposing models of N-tuple helixes: In addition to the three helixes—government, business, and university—they have also included the helix of (public) civil society and the helix of the natural environment.

In this way, the triple helix model was first transformed into a quadruple model through a public subsystem based on media and culture (Carayannis and Campbell, 2010; De Oliveira Monteiro and Carayannis, 2017). The purpose of this extension is to include the public and civil society as a fourth subsystem. Media-based audiences support not only the dissemination of knowledge but also the dissemination of culture with its values, experiences, traditions, and visions for the promotion of the knowledge society (Carayannis and Campbell, 2009, p. 217–227).

The quintuple helix finally stresses the socio-ecological perspective of the natural environments of society. Social ecology focuses on the interaction, co-development, and co-evolution of society, and nature. The goal of the quintuple helix is to include the natural environment as a new subsystem for knowledge and innovation so that “nature” becomes established as a central and equivalent component *of* and *for* knowledge production and innovation. “The natural environment for the process of knowledge production, and the creation of a new innovation is particularly important because it serves for the preservation, survival, and vitalization of humanity, and the possible making of new green technologies; and humankind, after all, should learn more from nature (especially in times of climate change)” (Carayannis et al., 2012, p. 5).

To analyze sustainability in a quintuple helix and to make sustainable development determination for progress, therefore, means that each of the five described helices has a special and necessary asset at its disposal, with a social (societal) and academic (scientific) relevance for use as follows:

(a) The education system (U): This first subsystem defines itself in reference to “academia,” “universities,” “higher education systems,” and schools. In this helix, the necessary “human capital” of a state is formed by the diffusion and research of knowledge;

(b) The economic system (I): As the second subsystem, this consists of “industry/industries and focuses”, “firms”, services, and banks. This helix concentrates on the “economic capital” of a state;

(c) The natural environment (E): It is the third subsystem, and it is decisive for sustainable development and provides people with a “natural capital”;

(d) The media-based and culture-based public (P): The fourth subsystem integrates and combines two forms of “capital”. On the one hand, this helix has, through the culture-based public (tradition, values, etc.), a “social capital”. On the other hand, the helix of the media-based public (television, the internet, newspapers, etc.) also contains “capital of information” (news, communication, and social networks);

(e) The political system (G): This is the fifth subsystem that is also of crucial importance because it formulates the “will”, where the state is heading in the present and future, thereby also defining, organizing as well as administering the general conditions of the state. Therefore, this helix has a “political and legal capital” (ideas, laws, plans, politicians, etc.) (see Carayannis et al., 2012, p. 5–6).

A system with five helixes is not linear; it is a web of interrelationships, different systems, niches, and paths that come together to sustain life. This new model extends the traditional linear chain model to the innovation process and broadens it to incorporate all aspects of society, academia, government, industry, public, and natural environment, thus creating a comprehensive National Ecological System of Innovation.

With this new framework, the focus remains on the organizational level, metrics, and measurement tools. To fully understand the innovation process, it is important to focus on interaction and relationships. People, organizations, and knowledge institutions rarely innovate alone, and innovation emanates from cumulative processes of interactive learning and research.

This means that the system must simultaneously refer to its elements and the relationships between these elements. Relationships may be seen as carriers of knowledge and interaction as processes in which new knowledge is produced and disseminated.

The key issue facing many organizations face is how they can promote effective innovation using organizational support mechanisms. A theoretical integration is needed to link organizational context with innovation and to consider strategic future orientation as an important action parameter for decisions about innovation and change. For these reasons

1. Organizations play the most important role in the innovation system;

2. Organizations innovate in interaction with other organizations and with the knowledge infrastructure;

3. The ways organizations innovate and learn reflect national innovation systems; and

4. Organizations belonging to different sectors contribute differently to innovation processes.

Based on these statements, the thesis that arises here is how the quintuple helix model can be a paradigm of future studies and technology foresight in the systemic attempt to observe the future of science, technology, society, economy, and their interactions in order to promote social, economic, and environmental benefits. From a social perspective, the model could provide guidance to improve the anticipatory profile of organizations and communities, helping to understand in a short time what the consequences of the present actions will be. From the point of view of technological application, the challenge is in understanding possible technological developments and in outlining possible economic, social, and market impact scenarios. In particular, the misuse of technology, the digitization of many living and learning environments, and the collection, management, and analysis of huge data raise many ethical and social issues; concerns about data security and privacy have been highlighted, as well as the more substantive issues about the influence and control that these technologies can have on people's lives. These risks can undermine citizens' confidence in these technologies and in the institutions and companies that use them (Amaturo et al., 2022, p. 636).

To support our thesis, some considerations concerning the defining aspects of this field of investigation are necessary.

It may be useful to start between technological forecasting and technology foresight, considering the following as the most representative:

1. Technological forecasting is the probabilistic estimate of future technological transfers and their consequences in the various technological and non-technological fields (Jantsch, 1967);
2. Technology forecasting concerns the creation of new technologies and the incremental and/or discontinuous change in existing technologies (Porter et al., 1991); and
3. Technology forecasting has been defined as a process of data collection and analysis aimed at predicting the future characteristics of useful techniques, machines, and procedures (Shillito and De Marle, 1992).

As Arnaldi (2012, p. 122–123) explains, reading these three definitions allows us to highlight some main characteristics of technological forecasting, such as the centrality of dynamics within the technical–scientific field in terms of the creation and transformation of technical devices and systems; the importance of assessing the economic, political, and social consequences of new technologies and their diffusion; the formalization of predictions relating to science and technology, to be understood in terms of probabilistic statements relating to the future, with a relatively high level of confidence.

These characteristics highlight two aspects related to technological forecasting. The first tells us how the relationship between scientific progress and technological development, in contrast, and society, on the other, remains anchored to an idea of the social impact of technology whose development is mainly traced back to internal dynamics of the techno-scientific sphere and its impacts are largely understood according to the essentially technology-driven vision; in many analyses of technology transfer, many aspects that are not directly internal (such as environment, social systems, and society) are defined in terms of consequences of technology transfer and not considered *a priori*.

Furthermore, traditional forecasting has the ambition to anticipate the most probable evolutionary paths of technologies and their impacts, assuming that this possible future can be connected in a linear way to the present. Otherwise, foresight attempts to overcome the typical rigidities of conventional forecasting and, therefore, constitutes an important evolution of technological forecasting. Some more recurring definitions of foresight tell us that:

1. It has been defined as the systematic attempt to observe the future of science, technology, economy, and society in an attempt to promote social, economic, and environmental benefits (Tegart, 2003);
2. It is the systematic process aimed at exploring the future in science, technology, and the economy of society in order to identify strategic areas for research and enabling technologies that offer the greatest probability of producing the greatest economic and social benefit (Martin, 1995); and
3. It is a process aimed at a better understanding of the forces that shape the long-term future that should be considered in policymaking, planning, and decision-making (Martin, 2010).

At this point, the characteristics that distinguish technology foresight from technological forecasting appear clearer. First, the process dimension of forecasting is emphasized: Not so much the formulation of a probabilistic statement as a learning process related to the future constitutes the heart of the foresight approach.

Second, foresight does not aim to anticipate evolutionary courses that are more probable but tries to systematically explore the alternative futures that emerge from the different possible configurations of factors and choices in the present. Finally, “this connection between anticipation and decision together with the recognition of the complexity of the interactions between different technical-scientific and social spheres justifies an effort of consultation and interaction between the scientific community, users of the innovation and decision-makers, both for cognitive purposes - through the use of interactive and participatory methods to produce anticipations - and of mobilization - to encourage the construction of new social networks capable of operating according to the identified visions and making the choices envisaged” (Arnaldi, 2012, p. 124, *my translation*).

**Figure 3:**
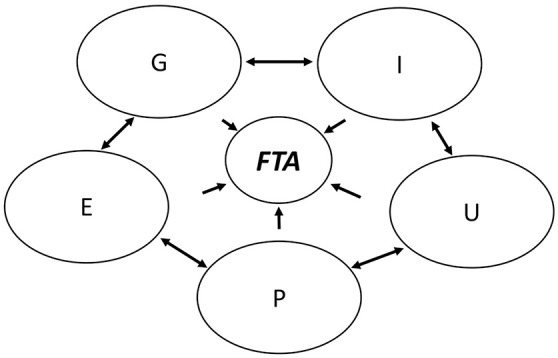
A quintuple helix model for foresight. Source: author's calculation. G, the political system; U, the education system; I, the economic system; P, the media-based and culture-based public; E, the natural environment; FTA, future-oriented technology analysis.

Hence, then, the opportunity to translate these observations into the quintuple helix model, as follows (Figure 3):

The systemic structure of the model, its internal dynamism, the monitoring action, and the feedback mechanism—without which the same general system is missing, so much so that it speaks of the *teleonomy of the system* (Paparella, 2014)—constitute the fundamental prerequisites for talking about spaces of foresight, to find stable systemic solutions to very complex situations (Dubois, 2000; Leydesdorff, 2008, 2021) and to implement *Future-Oriented Technology Analysis* (FTA).

In particular, we can expand the roles and functions of the previously identified helices as follows:

1. University (U): Develop new methodologies to reduce uncertainty in prospective; develop social foresight but also technological forecasting and technology foresight approaches;
2. Government (G): Make science and innovation policy more rigorous, transparent, and scalable; develop a technology assessment (TA) that is an analytic and democratic practice that aims to contribute to the formation of public and political opinion on societal consequences of science and technology;
3. Industries (I): Establish better connections and communications in order to accelerate innovations based on academic research; the public reaction and adoption matrix of innovative products should be useful in designing pathways for future innovation in products/processes (technology foresight);
4. Public (P): Highlight new bottom-up discoveries and innovations that improve social welfare, for example, eco-innovation; in addition, the media-based public supports not only the diffusion of knowledge in a state but also the culture-based public with its values, experience, traditions, and visions, which promotes knowledge; and
5. Environment (E): For the process of knowledge production, foresight on the natural environment is particularly important because it serves for the preservation, survival, and vitalization of humanity and the possible making of new green technologies (Chouaibi et al., 2022).

In this new configuration, the importance of TA should be underlined which is never just a tool or a specific type of policy analysis but is also part of the technology management policy in society, so much so that under the broad umbrella of TA there are also activities that go beyond simple analysis and instead structure part of the processes (Schot and Rip, 1996).

The recognition of the complex interactions between techno-science and society has meant that the object of the evaluation activities has changed over time, passing from the centrality of the technological element, of a specific technology, of a group of technologies to later problems related to the whole context of innovation. “The integration with the institutional processes of technical-scientific decision, the emphasis on the social context of technological innovation, the adoption of a more sophisticated vision of the relations between technology, science and society have supported the progressive distancing of the TA from its traditional expert-based version, toward a greater involvement of stakeholders and the public in the evaluation process. Definitions such as participatory TA, constructive TA, interactive TA share the idea of influencing the innovation process by promoting the action of citizens and stakeholders in the technological decision and including from the beginning the social aspects in the design of the technology thanks to an interaction timely with users, suppliers and various interested public” (Arnaldi, 2012, p. 126, *my translation*).

If foresight has always been understood as a tool to support national innovation systems, today TA is also increasingly oriented toward seizing the opportunities of future technological developments rather than preventing their undesirable effects.

But in any case, foresight and TA respond to the same questions and the same social needs and there is no reason not to believe that the typical sociability of the interaction space between development actors (helices) can translate into a vast field of anticipatory analysis of technology, that is to say, a *Future-oriented Tecnology Analysis* (FTA) based on knowledge, consensus and innovation spaces with some specific goals:

1. Orientation to the future without considering it predetermined;
2. The participation and enhancement of a plurality of actors;
3. In-depth knowledge of the phenomena, which also allows us to evaluate the consequences of the chosen paths;
4. Multidisciplinarity, for understanding the social and technical systemic complexity; and
5. The orientation to action: from forecasting based on historical data to a vision capable of anticipating changes.

## 5. Conclusions: Research limitations/implications

The great advantage of a multi-helix model is that it makes sociability possible (Simmel, 1983) which, as Ardigò recalls, is “a sort of cunning of reason that pushes humans to open up to others, to continuously intertwine reciprocal actions and communications” (Ardigò, 1988, p. 46, *my translation*).

Sociability makes the creation of a virtuous circle of development and social wellbeing possible: “facilitating the spread of a climate of trust and collaboration makes easier to create new collaborative forms for the socialization of knowledge, intelligence and technology transfer processes typical of a territory, with the natural consequence of foreseeing possible future scenarios and having a significant impact on the processes of generation and accumulation of intellectual capital, innovation and competitiveness. This type of relational governance favors collective learning forms and to make easier the economic and cultural exchanges necessary to the development and the social inclusion” (Martini, 2011, p. 137, *my translation*). However, there are also critical observations. If from a theoretical perspective, the not-centric dimension is guaranteed, the situation is the opposite in a practice plan. It is possible to say that the university covers an important detail in the model: It is considered the main source of production of scientific knowledge, and it has enormous responsibilities in terms of the spread and circulation of knowledge.

In this way, the university transfigures itself toward a model that is defined Hybrid University (Etzkowitz and Leydesdoff, 2001), assuming entrepreneurial nature and strengthening the relationship with the system of the enterprises: So that the third mission of the university is to contribute to economic development and social wellbeing, through technology transfer processes but above all through social and technological forecasting approaches (Etzkowitz, 2004; Etzkowitz and Viale, 2010).

If this is true, the technology forecasting policy could be considered the tool to promote sustainable and ethically responsible social construction processes of knowledge and innovation.

However, the technological transfer can represent a tool of application of the triple helix when in the generation of an innovative enterprise (product, service, process) technical–scientific feasibility; economic–financial feasibility, and government financial support are realized at the same time, that is with the active presence of scientific talents, managerial competences, and financial support.

For these reasons, it is important to observe the technological transfer and technology forecasting from a sociological point of view, and this finds an obvious justification in the growing attention that social dynamics are assuming in the possibilities of scientific production and technological application. Cultural, organizational, and economic differences require the ability to establish relationships between partners based on the need to imagine scenarios and consequences of actions, on the perception of fairness in exchanges, on the goal of generating a mutual profit and, therefore, useful to all parties involved. “In fact, is now famous the tendency of the research to develop itself through the involvement of more and more complex nets of actors. These favor the processes of innovation and valorization of the existing social and scientific relations, and that support the formations of new relations among subjects not gotten used to enter relationship among them. To this is joined that the network organizational system favors the sharing and the transfer of scientific and technological knowledge and concurs a more effective trade exploitation of the scientific discoveries and a wider spreading of the research output” (Martini and De Luca Picione, 2022, p. 443).

In this perspective, the role of a fourth and a fifth actor is inserted: the public and the natural environment. In their comparisons is necessary to reflect on the problem of the credibility of what to transfer, on the quality ethics of the involved actors, on the sustainability (environmental, social, and economic) of the modalities with which it is moved and, therefore, on the format to use in order to lead to good outcome the innovative communication. The convictions of Leydesdorff and Etzkowitz reside in the fact that the triple helix model was repeatedly subjected to ‘experiments': with the aid of “relevant data” they demonstrate the success or otherwise of the applicability of the model in one or more local contexts. They are not opposed *a priori* to N-tuples evolution, but they require effort to demonstrate how society and nature can initiate and support models of the quintuple helix.

In a discussion that focused on bringing “society” or “the public” back into the model as a fourth helix, Etzkowitz and Leydesdoff (2003) argued that the helices represent specialization and codification in function systems that evolve from and within civil society. As Leydesdorff (2013) recalls, a multifaceted society is no longer coordinated by a central nucleus but functions in terms of interactions between variously coded communications. Money, for example, can be considered an excellent example of a symbolically generalized means of communication (Parsons, 1968): It allows us to pay without having to negotiate the price of a commodity. Power, truth, trust, and affection are other “performative” media (Luhmann, 1995). Therefore, in a knowledge-based economy, one should not only optimize the conservation of “wealth from knowledge” but also support the generation of new research questions starting from social and economic needs. Variety is required in the different dimensions of triple or N-tuple helices so that differently coded discourses can select upon each other and interact (Ashby, 1958). One may wish to move beyond the triple helix model with three relevant selection environments, but every further dimension requires substantive specification, operationalization in terms of potentially relevant data, and sometimes the further development of relevant indicators (Leydesdorff and Sun, 2009).

The answer to Carayannis on the issue is that development must be understood within a systemic framework that cannot be separated from the role of society and the natural environment. “The Quintuple Helix innovation model (Carayannis and Campbell, 2010) bridges social ecology with knowledge production and innovation. Here, the natural environments of society and economy not only challenge, but also encourage and inspire knowledge production and innovation. In the approach of the Quintuple Helix innovation model, the ‘natural environments-of-society' are being identified as opportunities for driving further and excelling the sustainable development and coevolution of knowledge economy, knowledge society, and knowledge democracy” (Carayannis et al., 2012, p. 9).

Therefore, in this context, only a complete and solid “innovation ecosystem” will be able to guarantee that entrepreneurial drive and that capacity for innovation necessary to continue to compete successfully on international markets and help stimulate economic growth. The objective is essential to ensure the development of “real forms of reciprocity of social capital and not of a general willingness to cooperate and trust” (Pizzorno, 1999, p. 381, *my translation*).

Thanks also to the contribution of Science and Technology Studies, all the observations made so far on the effectiveness of the quintuple helix model underline the need to integrate not only technological innovation into the reference context but, in a reflective perspective, the anticipatory analysis of technological innovation. To this must necessarily be added an “ethical evaluation of digital technology” which consists in overcoming the traditional linear logic of impact, applying the so-called paradigm of coevolution between science and technology, which recognizes the open and hybrid character of change, which is, therefore, not technological but socio-technical, that is to say resulting from the integration and mutual influence of the two spheres of social and techno-science.

The diffusion of ICT and the strong digitization of many socio-economic–cultural spaces challenges the researcher and society to reflect on the known implications of their development and their application (among the many, think of the digital divide or, in the worst cases, technological drift) but also to be cautious about unforeseen and not yet known consequences (in these cases, many refer to the precautionary principle) (Buffardi, 2020). Indeed, it is clear that scientific and technological developments necessarily produce unintended consequences, which are very often the result of collective decisions rather than individual actions (Martini and Vespasiano, 2017, p. 71–72); “and it is not sufficient to build an ethics of science and technology on the basis of the image of a scientist who intentionally wants to create a Frankenstein. Rather, an ethical structure is required that addresses both the aspect of unintentional collateral consequences (as well as evidently the intentional ones) and that of collective and not just individual decisions” (von Schomberg, 2007, p. 5).^9^ Thus, this is even more true if we consider that in a world of “technical apparatuses” the ontological reversal between means and ends, subject and object takes place^10^: Man finds himself being the means, a raw material, for the indefinite perpetuation of the technical-economic development and, therefore, he becomes the medium between the world of apparatuses and the natural world.^11^ From a philosophical point of view, this leads the technique to become the subject of history, to the point that the technological world is transformed into a de-ideologized world. Consider, for example, also the concept of experience, which in this vision undergoes a profound modification: “if experience is the result of a conceptual elaboration of sensible data, to quote Kant, the technique has intervened by modifying the conceptual structures and the limits of our sensitive perception and our imagination. It is the problem of the supra-liminal that is to say the discrepancy between our imagining and representing the world and our producing by provoking it” (De Stefano, 2013, p. 117, *my translation*).

The awareness and acceptance of the mutual techno-science relationship make it possible to adopt a dynamic approach to exploring the interaction between morality and new technological solutions, thus making it possible to construct evolutionary scenarios of possible techno-scientific controversies, highlighting both the reasons for the possible arising of a dispute but also the processes that can lead to its closure.^12^

Predict, discover, and anticipate united in active participation, communication, knowledge, and action become so essential in the processes of production, as in the past it was the accumulation of capital, and also the ethical sensitivity begins to play an increasingly critical role. The most pressing question, then, is to re-examine the issues of efficiency and social equity, shifting the focus on individual freedoms (Sen, 1990), if you want to manage knowledge in an intelligent manner to the benefit of all (Martini, 2011, p. 207–208).

Probably, the usual planning tools are no longer enough: New tools are needed, and a different mindset, in particular, is needed. It is necessary to have the ability to develop scenarios and create anticipatory strategies in companies, in public administration, in the third sector, and in society as a whole.

Thus, we need to promote a real *Future Literacy* that responds to the need to transform human governance to use the future more efficiently. It is not just about how to prepare for potential crises or how to overcome great challenges: It is about moving beyond the dependence on the certainty illusions and the fragility that this certainty creates.

## Data availability statement

The original contributions presented in the study are included in the article/supplementary material, further inquiries can be directed to the corresponding author.

## Author contributions

The author confirms sole responsibility for the study conception and design, analysis and interpretation of bibliographic sources, and manuscript preparation.

## Footnotes

^1^“This conjunction between knowledge, imagination and will, goals and dreams, is frequently found in the thinking of intellectuals in developing countries. For example Concheiro (1984), inspired by the poets and scientists of the past, believes that society can be described not only through its history, but also through its projects. This way of reasoning and adapting by reflecting on the future, will be necessary for men tomorrow more than today, when they will be forced to live in a world of ever more rapid and interdependent changes” (Barbieri Masini, 2012, p. 15, *my translation*).

^2^In Latin language.

^3^For more information, see the studies on latents, including Rummel (1975–81) and Bloch (2005).

^4^For further information, see Poli (2012, p. 23–35).

^5^For the English version see: Working with the future. Ideas and tools to govern uncertainty (2019). Milano: Bocconi University Press.

^6^The point is that the nature of the window does not depend only on the technical properties of the model but also on the phenomenon being observed. What is needed is a window long enough to bring out significant changes: A shorter window fails to bring out the changes underway, and one that is too long overlaps the risk of confusing different cycles of change.

^7^In the text *Incertezza, futuro, narrazione* (2020), the author Emiliana Mangone addresses some issues that emerged in an emergency form with the COVID-19 pandemic, such as the perception of risk and the future, communication and storytelling, responsibility, etc. (Mangone, 2020).

^8^Relationships between subjects trigger a spiral of development, the dynamics of which imply a “vertical evolution and horizontal circulation”. “The extension of bilateral university-industry relations to trilateral university-industry-government relations was essential, since the model of a hypercycle is only meaningful in the case of three (or more) subdynamics. The hypercycle provides ametaphor for the supra-individual dynamics that give intersubjective meaning to the meanings provided by the carrying cycles. In other words, the emerging next-order-level ‘overlay' can contain a meta-representation of the individual representations and their interactions. This meta-representation in the hypercycle feeds back as a regime on the underlying dynamics which evolve historically along trajectorie” (Leydesdorff, 2021, p. 23–24).

^9^In this regard, Beck's reflection is interesting when he speaks of unaware or unknown non-knowledge and intentional non-knowledge, considered as the “cause” of the threat to humanity (Beck, 2008, p. 190 et seq.).

^10^Even in the reflections of Arthur (2009), the autonomous character of new technologies emerges, understood as combinations increasingly similar to something organic rather than mechanical (even if their ability to act is not equivalent to the freedom to choose that action: Freedom, in fact, provides that all the alternatives are co-present and that they possess an attraction, such as to induce the definitive choice).

^11^Consider that processes are currently being studied on how to “build computational architectures inspired by the human model, that is to say networks of silicon electronic components organized however as brain architectures”. It is evident that in this perspective *the future is biomimetic* (Cingolani, 2014, p. 74–75).

^12^A particular case of ethical technology assessment is the Vision Assessment, “the evaluation of visions” on future technological innovations. The Vision Assessment considers the visions themselves as a problem of ethical evaluation, which in this case does not concern the impacts of a present or future technology but the implicit and explicit assumptions about the present that the visions carry (Grunwald, 2007). Indeed, the persuasive capacity of visions makes them an important tool available to the various social actors to guide innovation processes. “These visions must be evaluated cognitively and ethically for their content and their evaluation must place the visionary anticipations within a series of alternative futures, the comparison of which helps to bring out the scientific hypotheses and assumptions underlying them, the social actors who are protagonists and those who are excluded, in order to make them the subject of a transparent democratic debate” (Arnaldi, 2012, p. 129*, my translation*).

## References

[B1] Amaturo E. Aragona B. Felaco C. (2022). Epistemic orientation for the technological future. Toward a research agenda for dealing with social challenges of internet of things. Ital. Sociol. Rev. 12, 635–649. 10.13136/isr.v12i7S.574

[B2] Ardigò A. (1988). Per una Sociologia Oltre il Post-Moderno. Roma-Bari: Laterza.

[B3] Arnaldi S. (2012). “Anticipazione e scelte tecnologiche,” in La Previsione Sociale. Introduzione allo Studio dei Futuri, eds S. Arnaldi, and R. Poli (Roma: Carocci), 121–132.

[B4] Arnaldi S. Poli R. (2012). La Previsione Sociale. Introduzione Allo Studio dei Futuri. Roma: Carocci.

[B5] Arthur B. W. (2009). The Nature of Technology: What It Is and How It Evolves. London: The Free Press and Penguin Book.

[B6] Ashby W. R. (1958). Requisite variety and its implications for the control of complex systems. Cybernetica 1, 1–17.

[B7] Bagnasco A. (2006). Imprenditorialità e capitale sociale: il tema dello sviluppo locale. Stato Mercato 78, 403–425. 10.1425/23231

[B8] Barbieri Masini E. (1982). Reconceptualizing furues: a need and a hope. World Fut. Bull. 6, 1–8.

[B9] Barbieri Masini E. (1994). The Futures of Cultures. Paris: UNESCO.

[B10] Barbieri Masini E. (2012). “Introduzione. Perchè pensare al future oggi?,” in La Previsione Sociale. Introduzione Allo Studio dei Futuri, eds S. Arnaldi, and R. Poli (Roma: Carocci), 13–22.

[B11] Beck U. (2008). Conditio Humana. Il rischio nell'età globale. Roma-Bari: Latwrza (original edition 2007, Weltrisikogesellschaft. Auf der Suche nach der verlorenen Sicherheit).

[B12] Bell W. (2003). Foundations of Futures Studies. Volume 1: History, Purposes, and Knowledge. London: Transaction Publishers.

[B13] Berger G. (1964). Phénoménologie du Tempest Prospective. Paris: PUF.

[B14] Berthoz A. (2004). La Decisione. Torino: Codice.

[B15] Bloch E. (2005). Il Principio Speranza. Milano: Garzanti.

[B16] Buffardi A. (2020). Futuri Possibili. Formazione, Innovazione, Culture Digitali. Milano: Egea.

[B17] Cagning G. Keenan M. (2008). “Positioning future-oriented technology analysis,” in Future-Oriented Technology Analysis: Strategic Intelligence for an Innovative Economy, eds G. Cagning, M. Keenan, F. Scapolo, and R. Barré (Berlin: Springer), 1–13.

[B18] Calderini M. (2005). Una Tripla Elica per rilanciare lo sviluppo. La Stampa 135, 28.

[B19] Carayannis E. G. Barth T. D. Campbell D. F. (2012). The Quintuple Helix innovation model: global warming as a challenge and driver for innovation. J. Innovat. Entrepreneur. 1, 2. 10.1186/2192-5372-1-2

[B20] Carayannis E. G. Campbell D. F. J. (2009). ‘Mode 3' and ‘Quadruple Helix': toward a 21st century fractal innovation ecosystem. Int. J. Technol. Manag. 46, 201–234. 10.1504/IJTM.2009.023374

[B21] Carayannis E. G. Campbell D. F. J. (2010). Triple helix, quadruple helix and quintuple helix and how do knowledge, innovation, and environment relate to each other? Int. J. Soc. Ecol. Sustain. Dev. 1, 41–69 10.4018/jsesd.2010010105

[B22] Carayannis E. G. Campbell D. F. J. (2012). Mode 3 knowledge production in quadruple helix innovation systems. 21st-century democracy, innovation, and entrepreneurship for development. Springer Briefs Bus. 7, 1–63. 10.1007/978-1-4614-2062-0_1

[B23] Carayannis E. G. Campbell D. F. J. Rehman S. S. (2016). Mode 3 knowledge production: systems and systems theory, clusters and networks. J. Innovat. Entrepreneur. 17, 1–24 10.1186/s13731-016-0045-9

[B24] Carayannis E. G. Formica P. (2006). Intellectual venture capitalists: an emerging breed of knowledge entrepreneurs. Ind. High. Educ. 20, 151–156. 10.5367/000000006777691034

[B25] Chouaibi S. Chouaibi J. Rossi M. (2022). ESG and corporate financial performance: the mediating role of green innovation: UK common law versus Germany civil law. Euromed J. Bus. 17, 46–71. 10.1108/EMJB-09-2020-0101

[B26] Cingolani R. (2014). Il mondo è Piccolo Come Un'arancia. Una Discussione Semplice Sulle Nanotecnologie. Milano: il Saggiatore.

[B27] Concheiro A. A. (1984). Refleciones Sobre Prospective. México: Centro de Estudios Prospectivos de la Fundación Javier Barros Sierra.

[B28] Cournand A. Lévy M. (1973). Shaping the Future: Gaston Berger and the Concept of Prospective. New York, NY: Gordon and Breach Science Publishers.

[B29] De Jouvenel B. (1967). The Art of Conjecture. London: Weidenfeld and Nicholson.

[B30] De Oliveira Monteiro S. P. Carayannis E. G. (eds). (2017). The Quadruple Innovation Helix Nexus. A Smart Growth Model, Quantitative Empirical Validation and Operationalization for OECD Countries. New York, NY: Palgrave Macmillan.

[B31] De Stefano L. (2013). La libertà fragile. Una prospettiva antropologica tra Günther Anders e Andrè Leroi-Gourhan. Sci. Filosofia 9, 115–139.

[B32] Derbyshire J. Wright G. (2017). Augmenting the intuitive logics scenario planning method for a more comprehensive analysis of causation. Int. J. Forecast. 33, 254–266. 10.1016/j.ijforecast.2016.01.004

[B33] Dubois D. M. (2000). “Review of incursive, hyperincursive and anticipatory systems - foundation of anticipation in electromagnetism,” in Computing Anticipatory Systems: CASYS'99 - Third International Conference, AIP Conference Proceedings 517, ed Id (Melville: The American Institute of Physics), 3–30.

[B34] Etzkowitz H. (2004). The evolution of the entrepreneurial university. Int. J. Technol. Globalis. 1, 64–77. 10.1504/IJTG.2004.004551

[B35] Etzkowitz H. (2008). The Triple Helix: University-Industry-Government Innovation in Action. New York, NY: Routledge.

[B36] Etzkowitz H. Leydesdoff L. (1995). The triple helix university-industry-government relations: a laboratory for knowledge based economic development. EASST Rev. 14, 14–19.

[B37] Etzkowitz H. Leydesdoff L. (1998). The triple helix as a model for innovation studies. Sci. Public Policy 25, 195–203.

[B38] Etzkowitz H. Leydesdoff L. (2000). The dynamics of innovation: from National Systems and “Mode2” to a Triple Helix of university-industry-government relations. Res. Policy 29, 109–123. 10.1016/S0048-7333(99)00055-4

[B39] Etzkowitz H. Leydesdoff L. (2001). The transformation of university-industry-government relations. Electron. J. Sociol. 5, 338–344.

[B40] Etzkowitz H. Leydesdoff L. (2003). Can ‘the public' be considered as a fourth helix in university-industry-government relations? Sci. Public Policy 30, 55–61 10.3152/147154303781780678

[B41] Etzkowitz H. Viale R. (2010). Polyvalent knowledge and the entrepreneurial university: a third academic evolution? Crit. Sociol. 36, 4. 10.1177/0896920510365921

[B42] Facioni C. (2019). Per una Sociologia dei Futuri: Il Contributo di Eleonora Barbieri Masini Alla Fondazione dei Futures Studies. Naples: Futuri, VI, Italian Institute for the Future, 65–86.

[B43] Flechtheim O. K. (1943). “Futurologie,” in Historiches Worterbuch der Philosophie, ed J. Ritter (Basel: Schwabe & Co.).

[B44] Freeman C. (1987). Technology and Economic Performance: Lessons from Japan. London: Pinter.

[B45] Freeman C. (1995). The national innovation systems in historical perspective. Cambrid. J. Econ. 19, 5–24.

[B46] Grunwald A. (2007). Converging technologies: visions, increased contingencies of the conditio humana, and search for orientation. Futures 4, 380–392. 10.1016/j.futures.2006.08.001

[B47] Grupp H. Linstone H. A. (1999). National technology foresight activities around the globe: resurrection and new paradigms. Technol. Forecast. Soc. Change 60, 85–94 10.1016/S0040-1625(98)00039-0

[B48] Hatakeyama K. Ruppel D. (2004). “Sabato's triangle and international academic cooperation: the importance of extra-relations for the Latin American enhancement,” in Progress Through Partnership (International Conference on Engineering Education and Research. VSB-TUO) (Ostrava), 535–539.

[B49] Jantsch E. (1967). Technological Forecasting in Perspective. Paris: OECD.

[B50] Kothari R. (1975). Footsteps into the Future: Diagnosis of the Present World and a Design for an Alternative. New York, NY: The Free Press.

[B51] Kurzweil R. (2005). The Singularity is Near: When Humans Transcend Biology. New York, NY: Viking Press.

[B52] Leydesdorff L. (2005). The triple helix model and the study of knowledge-based innovation systems. Int. J. Contemp. Sociol. 42, 12–27.

[B53] Leydesdorff L. (2006). The Knowledge-Based Economy. Modeled, Measured, Simulated. Irvine, CA: Universal-Publishers.

[B54] Leydesdorff L. (2008). “The communication of the meaning in anticipatory system: a simulation study of the dynamics of intentionality in social interaction,” in Proceedings of the 8th International Conference on Computing Anticipatory Systems, ed D. M. Dubois (Melville: The American Institute of Phisycs), 33–49.

[B55] Leydesdorff L. (2010). The knowledge-based economy and the triple helix model. Ann. Rev. Inf. Sci. Technol. 44, 365–417. 10.1002/aris.2010.144044011628232771

[B56] Leydesdorff L. (2013). “Triple helix of university-industry-government relations,” in Encyclopedia of Creativity, Innovation, and Entrepreneurship, ed E. G. Carayannis (New York, NY: Springer), 1844–1851.30735551

[B57] Leydesdorff L. (2021). The Evolutionary Dynamics of Discursive Knowledge: Communication-Theoretical Perspectives on an Empirical Philosophy of Science. Cham: Springer Nature.

[B58] Leydesdorff L. Sun Y. (2009). National and international dimensions of the triple helix in Japan: university industry-government versus international co-authorship relations. J. Am. Soc. Inf. Sci. Technol. 60, 778–788. 10.1002/asi.20997

[B59] Livraghi R. (2003). Economia Della Conoscenza e Capitale Sociale. Quaderni di Economia del Lavoro. n. 76/77. Milano: FrancoAngeli.

[B60] Luhmann N. (1990). Sistemi Sociali. Fondamenti di una Teoria Generale. Bologna: il Mulino (original edition 1984, Soziale Systeme. Grundriß einer allgemeinen Theorie).

[B61] Luhmann N. (1995). Social Systems. Stanford, CA: Stanford University Press.

[B62] Lundvall B. Å. (1992). National Systems of Innovation: Towards a Theory of Innovation and Interactive Learning. London: Pinter Publishers.

[B63] Lundvall B. Å. (1998). Why study national systems and national styles of innovation? Technol. Anal. Strat. Manag. 10, 403–422. 10.1080/0953732980852432422436094

[B64] Mangone E. (2020). Incertezza, Futuro, Narrazione. Fisciano: NaSC Free Press.

[B65] Martin B. R. (1995). Foresight in science and technology. Tecnovation 31, 69–76 10.1080/09537329508524202

[B66] Martin B. R. (2010). The origins of the concept of ‘foresight' in science and technology: an insider's perspective. Technol. Forecast. Soc. Change 9, 1438–1447. 10.1016/j.techfore.2010.06.009

[B67] Martini E. (2011). Socializzare per Innovare. Il Modello Della Tripla Elica. Napoli: Loffredo.

[B68] Martini E. De Luca Picione R. (2022). “Triple helix model: a device for social construction of knowledge and innovation,” in Handbook of Research on Applying Emerging Technologies Across Multiple Disciplines, ed E. Marchisio (Pensylvania: Igi Global), 433–452.

[B69] Martini E. Vespasiano F. (2011). “The paradigm of the social construction of knowledge: the triple helix model,” in Essays on Social Themes. Athens Institute for Education and Research, ed G. T. Papanikos (Athens: ATINER), 175–190.

[B70] Martini E. Vespasiano F. (2015). “Territorial dynamics: the rules of innovation helices,” in Management Innovation, Entrepreneurship and Human Resource Management practices: A Global Perspective, eds D.Vrontis, G. Sakka, and M. Amirkhanpour (Newcastle upon Tyne: Cambridge Scholars Publishing), 75–91.

[B71] Martini E. Vespasiano F. (2017). Scienza con coscienza: la riflessività sociale, per un'etica del futuro. Stud. Sociol. 1, 65–79.

[B72] Paparella N. (2014). “A proposito di terza missione: una nuova versione del modello di tripla elica,” *Terza Missione. Parametro di Qualità del Sistema Universitario*, ed C. Formica (Napoli: Giapeto Editore), 11–38.

[B73] Parsons T. (1968). “Interaction: social I. Interaction,” in The International Encyclopedia of the Social Sciences, ed. D. L. Sills (New York, NY: McGraw-Hill), 429–441.

[B74] Pizzorno A. (1999). Perché si paga il benzinaio. Nota per una teoria del capitale sociale. Stato Mercato 57, 373–395.

[B75] Poli R. (2012). “Le basi teoriche della previsione sociale,” in La previsione sociale. Introduzione allo studio dei futuri, eds S. Arnaldi, and R. Poli (Roma: Carocci), 23–36.

[B76] Poli R. (2019). Lavorare con il futuro. Idee e strumenti per governare l'incertezza. Milano: Egea.

[B77] Poli R. (2020a). “Anticipation,” in The Palgrave Encyclopaedia of the Possible, ed V. Glaveanu (Switzerland: Springer), 1–7.

[B78] Poli R. (2020b). “Forecasting,” in The Palgrave Encyclopaedia of the Possible, ed V. Glaveanu (Switzerland: Springer), 1–6.

[B79] Poli R. (2020c). “Foresight,” in The Palgrave Encyclopaedia of the Possible, ed V. Glaveanu (Switzerland: Springer), 1–7.

[B80] Porter A. L. Roper T. Mason T. Rossini F. Banks J. (1991). Forecasting and Management of Technology. New York, NY: Wiley.

[B81] Riegler A. (2003). “Whose anticipations?,” in Anticipatory Behavior in Adaptive Learning Systems: Foundations, Theories, and Systems, eds M. Butz, O. Sigaud, and P. Gerard (Berlin: Springer-Verlag), 11–22.

[B82] Rosen R. (1985). Anticipatory Systems Philosophical, Mathematical, and Methodological Foundations. 2nd Edn. New York, NY: Springer.

[B83] Rummel R. J. (1975–81). Understanding Conglict and War. Vol. 5. New York, NY: Wiley and Sons.

[B84] Schot J. Rip A. (1996). The past and future of constructive technology assessment. Technol. Forecast. Soc. Change 54, 251–268. 10.1016/S0040-1625(96)00180-1

[B85] Schütz A. (1960). Der sinnhafte Aufbau der sozialen Welt. Wien: Springer-Verlag. (it. translation: La fenomenologia del mondo sociale. Bologna: Il Mulino 1974).

[B86] Sen A. (1990). Individual freedom as a social commitment. New York Rev. Books 37, 49–55.

[B87] Shillito M. De Marle D. J. (1992). Value: Its Measurement, Design, and Management. New York: Wiley.

[B88] Simmel G. (1983). Forme e giochi di società. Problemi fondamentali della società. Milano: Feltrinelli Socio Economic System. The IPTS Report 29, 1–8.

[B89] Tegart G. (2003). Technology foresight: philosophy and principles. Innovat. Manag. Policy Pract. 5, 279–285. 10.5172/impp.2003.5.2-3.27930289669

[B90] Tuomi I. (2019). Chronotopes of foresight: models of time-space in probabilistic, possibilistic and constructivist futures. Fut. Foresigt Sci. e11. 10.1002/ffo2.11

[B91] UNIDO (1999). Technology Foresight Manual. Wien: UNIDO.

[B92] Viale R. (2001). Ricerca e innovazione in Europa e Stati. Kéiron, 8. Available online at: http://www.farmindustria.it (accessed December 2010).

[B93] Viale R. Ghiglione B. (1998). The Triple Helix Model: A Tool for the study of European Regional. European Commission: The IPTS Report, 29, 1–8.

[B94] von Schomberg R. (2007). From the Ethics of Technology towards an Ethics of Knowledge Policy and Knowlwdge Assessment. European Commission, Directorate-General for Research and Innovation: Publications Office.

[B95] Yawson M. R. (2009). “The ecological system of innovation: a new architectural framework for a functional evidence-based platform for science and innovation policy,” in The Future of Innovation. Proceedings of XX ISPIM 2009 Conference, eds K. R. E. Huizingh, S. Conn, M. Torkkeli, and I. Bitran (Wien).

